# Conferring specificity on the ubiquitous Raf/MEK signalling pathway

**DOI:** 10.1038/sj.bjc.6601488

**Published:** 2004-01-20

**Authors:** E O'Neill, W Kolch

**Affiliations:** 1 Beatson Institute for Cancer Research, Switchback Road, Glasgow G61 1BD, UK; 2 Institute of Biomedical and Life Sciences, Sir Henry Wellcome Functional Genomics Facility, University of Glasgow, Glasgow G12 8QQ, UK

**Keywords:** Raf, MAPK, signalling, substrate, activation, isoforms

## Abstract

The Raf-MEK-ERK signalling pathway controls fundamental cellular processes including proliferation, differentiation and survival. It remains enigmatic how this pathway can reliably convert a myriad of extracellular stimuli in specific biological responses. Recent results have shown that the Raf family isoforms A-Raf, B-Raf and Raf-1 have different physiological functions. Here we review how Raf isozyme diversity contributes to the specification of functional diversity, in particular regarding the role of Raf isozymes in cancer.

Raf first came to the fore as a retroviral oncogene, v-Raf or v-Mil, which could induce tumours in mice and chickens, respectively. Raf-1, the cognate proto-oncogene, is widely expressed and became the most intensely studied Raf family member. The other two family members, A-Raf and B-Raf, feature a more restricted expression. A-Raf is mainly expressed in urogenital tissues and B-Raf expression is highest in neuronal tissues, testis and haematopoetic cells, although recent experiments suggest that the expression of A-Raf and B-Raf at low levels is much more widely spread (reviewed by Kolch, 2000).

Interest in Raf proteins surged upon identification of their role as direct effectors of Ras. Ras has suffered oncogenic mutations in nearly 30% of all human cancers. All the three Raf family members share the biochemical properties of binding to Ras and to phosphorylate and activate MEK (reviewed by Kolch, 2000; Avruch *et al*, 2001). Currently, MEK is the only generally acknowledged substrate of Raf kinases. MEK phosphorylates and activates ERK. While Raf and MEK appear restricted to only one class of substrates, ERK musters more than 70 substrates including nuclear transcription factors (Lewis *et al*, 1998). This led to the perception of a linear pathway where Ras funnels a great variety of extracellular cues into a three-tiered kinase module, Raf-MEK-ERK, which relies on ERK to dispense the signal to various substrates. An obvious problem with this view is how to rationalise that this pathway controls many and diverse fundamental cellular functions including proliferation, differentiation, transformation and apoptosis.

The specificity of biological responses can be encoded in a number of ways. Specificity can be achieved by compartmentalisation, where the accessibility to upstream activators and downstream substrates is regulated by subcellular localisation. The response can also depend on the cellular context, such as the expression of other proteins and activity of other pathways. This requires points of crosstalk where these contextual signals are integrated. In such a scenario, the exact kinetics and duration of ERK signalling will play a major role. Such fine tuning can be adjusted by the differential response of upstream activators to external cues. There may also be yet unrecognised branchpoints that distribute signals into other downstream pathways. These mechanisms are not mutually exclusive, and recent discoveries support all of these possibilities. In this review, we will briefly discuss this evidence.

## RAF GENE KNOCKOUTS

The most convincing case for isozyme-specific functions of the three Raf family genes was made by knockout studies in mice. The ablation of the A-raf gene (Pritchard *et al*, 1996) results in neurological defects. In an inbred background, mice die 7–21 days post partum from megacolon caused by a defect in visceral neurons that control bowel contractions. In an outbred background, A-raf^−/−^ mice survive to adulthood, but still feature defects in proprioception and abnormal movements. This phenotype resembles Neurotrophin-3 (NT-3)-deficient mice (Kirstein and Farinas, 2002). NT-3 also promotes the survival of visceral neurons. These observations would place A-Raf in a NT-3-mediated neuronal survival pathway. The lack of A-Raf did not affect the regulation of ERK in mouse embryonic fibroblasts (MEFs) or the transformation of these cells, suggesting that these functions are compensated for by the other Raf family members (Mercer *et al*, 2002).

Raf-1-deficient mice die in midgestation, due to widespread apoptosis throughout the embryo (Huser *et al*, 2001; Mikula *et al*, 2001). The penetrance of the phenotype was more severe in inbred than in outbred backgrounds. Surprisingly, ERK activation by growth factors was not compromised in Raf-1^−/−^ MEFs, presumably due to compensation by B-Raf. Even more surprisingly, a Raf-1 mutant that is refractory to growth factor stimulation could rescue the knockout phenotype, resulting in viable and apparently normal mice (Huser *et al*, 2001). Overall, the Raf-1^−/−^ phenotype resembles K-Ras^−/−^ as well as Fas-ligand transgenic mice. These observations suggest that Raf-1 is a main effector of K-Ras, and that a major task of Raf-1 is to restrain the proapoptotic function of Fas signalling. Indeed, Raf-1 MEFs exhibited increased sensitivity to Fas-induced apoptosis (Huser *et al*, 2001; Mikula *et al*, 2001). Much needed detailed mechanistic investigations are just beginning. The cause of the anaemia in Raf-1^−/−^ animals was traced back to the elevation of caspase-1 activity that accelerated erythroid differentiation to a point where erythroid progenitor cells became depleted (Kolbus *et al*, 2002). All these observations point to effectors distinct from MEK and ERK mediating Raf-1's antiapoptotic role.

B-raf^−/−^ mice (Wojnowski *et al*, 1997) provided the first genetic evidence for a role of a Raf isoform in the regulation of apoptosis. These mice die in midgestation, due to haemorrhage caused by massive apoptosis of endothelial cells. However, the interpretation of these data is confounded by the uncertainty whether the knockout strategy has abolished the expression of all the B-raf splice forms. Nevertheless, the very different phenotypes of the Raf knockout mice indicate that Raf isoforms have different biological functions. B-Raf emerges as the main regulator of the MEK-ERK pathway, while Raf-1 and A-Raf seem to provide ERK-independent apoptosis protection in different tissues. This conclusion is likely too simplistic, but can serve as a useful working hypothesis for the dissection of Raf isoform function.

An elegant biochemical study of the contribution of Raf-1 and B-Raf to B-cell receptor (BCR) signalling showed that Raf-1 and B-Raf are activated with different kinetics and cooperate in many downstream responses (Brummer *et al*, 2002). DT40 B-cells were engineered so that either gene could be deleted individually, or both together. Raf-1 was dispensable for BCR-mediated ERK activation, while the ablation of B-Raf caused a reduced and shortened activity of ERK. The Raf-1/B-raf double knockout resulted in an almost complete loss of ERK activation and severely reduced expression of the downstream transcriptional targets c-Fos and Egr-1. Only the activation of nuclear factor of activated T cells, NFAT, was mainly dependent on B-Raf. The molecular basis for the cooperation between Raf-1 and B-Raf is not yet understood. It could rely on complementation at the level of downstream substrates, including specific differences in activation kinetics. It also could be related to the ability of Raf-1 to heterodimerise with B-Raf. The heterodimer, conceivably, could have different signalling properties than either individual protein.

## RAF ISOFORM REGULATION AND FUNCTION

All the three Raf isoforms contain a Ras-binding site (RBD) in the regulatory domain at the N-terminus, and can consequently be activated by Ras GTPases. The RBD selectively binds to activated GTP-loaded Ras, but the effects are different. Ras binding does not activate Raf-1 directly, but seems to serve to translocate Raf-1 from the cytosol to the membrane, where subsequent activation events occur (Dhillon and Kolch, 2002). These comprise a complex series of events starting with the dephosphorylation of S259, which allows phosphorylation of S338 and possibly Y341 as well as two sites in the activation loop. These modifications work together not only to determine the quantity of the signalling output, but also maybe even the quality. Raf-1 S259 mutant can activate ERK to a similar extent as the v-Raf oncogene, yet fails to transform the cells, suggesting that the quality of the downstream signal is different depending on the upstream mode of activation (Dhillon *et al*, 2003). Rather little is known about the regulation of A-Raf, but it seems to be regulated in a similar way as Raf-1 yet binds to Ras more weakly and also is a weaker MEK kinase (Marais *et al*, 1997).

In contrast, B-Raf is activated directly by Ras binding. B-Raf also features a phosphomimetic aspartate in place of the residue corresponding to Y341, and is constitutively phosphorylated at the S338 equivalent (Mason *et al*, 1999). Thus, B-Raf appears to have shortcircuited several of the events required for the activation of Raf-1. This is consistent with B-Raf possessing a much higher specific activity towards MEK than Raf-1. This could explain why B-raf can fully compensate for the loss of Raf-1 or A-Raf in the knockout cells pertaining to the regulation of ERK.

**Figure 1:**
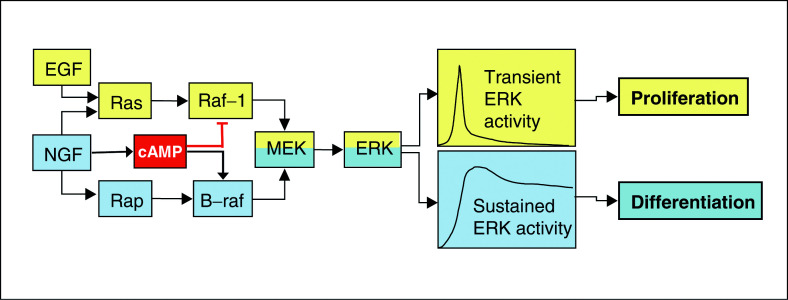
PC12 cell model of neuronal differentiation. This model shows how a biological response is specified by the kinetics and duration of ERK activity, which is achieved through the combinatorial integration of activating different Raf isoforms and crosstalk with the cAMP signalling system. PC12 cells differentiate in response to the nerve growth factor (NGF), but proliferate in response to the epidermal growth factor (EGF). Both growth factors utilise the Raf/MEK/ERK pathway. The biological response is determined by the duration of ERK signalling. Sustained ERK activation results in neuronal differentiation. The sustenance of ERK activity is caused by the B-Raf isoform, which is activated preferentially by NGF. Differentiation is further enhanced by activation of cAMP signalling, which inhibits Raf-1, but promotes B-Raf activity.

In addition, B-Raf can be activated by Rap1 (York *et al*, 1998), a Ras-related protein, which was originally described as a suppressor of Ras transformation. This inhibitory function seems to be due to the ability of Rap1 to sequester Raf-1 in inactive complexes. As this requires Rap1 overexpression, it is unclear whether this function of Rap1 is physiological. Rap1 is activated by numerous growth factors and appears to be a signal transducer in its own right (Bos *et al*, 2001). Again, it has been disputed whether B-Raf is a physiological Rap1 effector (Bos *et al*, 2001), but at least under certain conditions it can promote PC12 cell differentiation by activating B-Raf. The higher activity of B-Raf ensures the sustained activation of ERK that is required for neuronal differentiation of PC12 cells. However, there is also differential regulation downstream through crosstalk with the cAMP signalling system. The cAMP-dependent protein kinase PKA phosphorylates and inhibits Raf-1. In contrast, cAMP induces the activation of B-Raf by activation of Rap1 (York *et al*, 1998). Thus, cAMP should interfere with ERK activation in cells where only Raf-1 is expressed, but promote ERK activation in cells where B-Raf is coexpressed. Switching from one Raf isoform with low MEK kinase activity to another with high activity could serve to fine-tune the activation dynamics of ERK, in order to determine a specific biological response (Figure 1

). A recent study has shown how ERK activation kinetics can be converted into differential responses (Murphy *et al*, 2002). A transient activation of ERK will induce the expression of Fos by phosphorylating the Elk and Sap transcription factors. As the Fos protein is very unstable, the impact on a transcriptional response is limited. However, sustained ERK signalling results in the activation of Rsk, which phosphorylates and stabilises Fos, producing a robust transcriptional activation.

## RAF ISOFORMS IN CANCER

Raf-1 is the cellular proto-oncogene homologue of v-Raf. v-Raf corresponds to the Raf-1 kinase domain. Indeed, the deletion of the regulatory domain converts Raf-1 into an oncogene, termed BXB (Heidecker *et al*, 1990). However, genetic alterations of Raf-1 in human tumours have not been found. Lung tumour cell lines often overexpress Raf-1, and a transgenic mouse model has been developed where Raf gene expression is targeted to the lungs by use of a tissue-specific promoter (Rapp *et al*, 2003). BXB overexpression rapidly induced numerous well-differentiated, noninvasive adenomas. Surprisingly, the overexpression of Raf-1, which is nontransforming in cell culture models, also resulted in the formation of adenomas with the same histological phenotype. These tumours were fewer and delayed, and, in contrast to BXB, induced adenomas did not feature elevated ERK activity. These findings suggest that Raf-1 contributes to different aspects of malignancy, including ERK-independent processes and those which escape *in vitro* experimentation. Crossing the BXB transgenic mice with bcl-2 knockout mice did not change the tumour phenotype, but retarded tumour development mainly due to an increase in the rate of apoptosis. In contrast, the concomitant loss of p53 accelerated tumour growth and also changed the histological composition from cuboid cells to papillary tumours with large columnar epithelial cells. Despite occasional bronchiolar invasion, no metastasis was observed. These phenotypes resemble human atypical adenomatous lung hyperplasia, which may be the initial lesion on the way to lung adenocarcinoma (Mori *et al*, 2001). Thus, studies to validate the role of Raf-1 in human lung cancer are eagerly awaited. In particular, it may be interesting to examine the role of B-Raf.

B-Raf is mutated in 66% of melanomas and a smaller number of other prevalent cancers such as colon tumours. While the activation of Raf-1 requires a complex series of events, the activation of B-Raf is much simpler to achieve, explaining why B-Raf is a preferred target for mutational activation in human cancers (Mercer and Pritchard, 2003). The main mutation is V599E, which introduces a negative charge into the activation loop. This phosphomimetic mutation suffices to deregulate B-Raf activity and convert it into an oncogene (Davies *et al*, 2002). In the meantime, a flurry of studies has established that B-Raf is indeed mutated in a great variety of cancers albeit, with the exception of thyroid carcinoma, B-Raf mutation has a rather low incidence (Mercer and Pritchard, 2003). Most of these tumour types are known to have Ras mutations, but interestingly, Ras and B-Raf mutations usually do not coincide in the same tumour. This observation provides strong genetic evidence that B-Raf is a crucial effector for Ras-mediated tumourigenesis; however, the exact molecular mechanism is still obscure. Besides the prevalent V599E mutation, which enhances kinase activity, there are three other classes of mutations whose functional consequences are less clear (Mercer and Pritchard, 2003). One class affects the Akt phosphorylation sites in B-Raf, which have been implicated in the inhibition of B-raf kinase activity. Obviously, these mutations would only activate B-raf in cells where its activity is restrained through Akt. Another class of mutations that leads to modest activation is found in the ATP-binding glycine-rich loop. Yet another type alters the conserved DFG motif at the base of the activation loop. Such mutations usually incapacitate the kinase activity. These mutations are rare, but challenge the view that the oncogenic conversion of B-Raf is only due to the chronic hyperstimulation of the MEK-ERK pathway. A plausible explanation is that B-Raf has a role that is independent of its kinase activity, for instance, as a scaffolding protein or as a dominant-negative mutant, which sequesters proteins that prevent transformation.

## DIVERSIFICATION AT THE SUBSTRATE LEVEL

B-Raf appears to be the main activator of the MEK-ERK pathway (Huser *et al*, 2001; Mikula *et al*, 2001), and the simplicity of B-Raf activation rationalises why B-raf is a preferred target for oncogenic mutation. However, it also leaves a puzzle. Why does nature go to such great lengths to regulate the comparably poor kinase activity of Raf-1 in this very complicated manner? A provocative possibility is that MEK is not the main relevant substrate of Raf-1, or that these post-translational modifications serve as docking platforms to assemble functionally different Raf-1 complexes.

The interpretation of yet unknown Raf-1 substrates is in line with the phenotype of the Raf-1^−/−^ mice which succumb to deregulated apoptosis despite an apparently normal regulation of ERK. There is also a growing body of circumstantial evidence that not all functions of Raf-1 are dependent on its ability to activate MEK (Hindley and Kolch, 2002). Obviously, the existence of Raf isoform-specific substrates would neatly explain isoform diversity. A number of alternative Raf-1 substrates have been described, but none has been unequivocally validated yet. One presumably Raf isoform-specific substrate is the retinoblastoma protein (Rb). Rb phosphorylation is required to traverse the G1–S-phase boundary of the cell cycle. Although Rb is classically viewed as a target for cyclin D- and E-dependent cell cycle kinases, other kinases may contribute to its inactivation. Raf-1 has been reported to promote the inactivation of Rb by directly phosphorylating it. Rb phosphorylation was dependent on binding to the 25 N-terminal amino acids of Raf-1. This stretch is unique and hence Rb should be a Raf-1 isoform-specific target (Wang *et al*, 1998).

However, there is also the possibility that Raf isoforms convey specificity through conveying differential activation kinetics on their common substrate MEK. The PC12 cell paradigm has been discussed above. Studies in IL-3-dependent haematopoetic cell lines showed that the A-Raf kinase domain abrogates growth factor dependence more efficiently than the Raf-1 or B-Raf kinase domains. However, in all cases, this process was MEK dependent (Hoyle *et al*, 2000).

Yet another level of specificity could be achieved through the differential activation of MEK and ERK isoforms. Both MEK and ERK feature two isoforms in mammalian cells. A common assumption is that they are functionally equivalent. However, their evolutionary conservation suggests that they exert nonredundant functions. This is proven by the phenotype of MEK and ERK knockout mice. Knocking out MEK-1 results in an embryonic lethal phenotype which is similar, but not identical to the Raf-1 knockout (Giroux *et al*, 1999). This confirms that MEK-1 is a physiologically relevant target of Raf-1, but also points to a more complicated scenario where signals diversify from MEK isoforms. Indeed, in Hela cells, A-Raf selectively activated MEK-1 in response to EGF, whereas Raf-1 activated both MEK-1 and MEK-2 (Wu *et al*, 1996). In a similar vein, the ablation of ERK-1 does not compromise viability and causes rather subtle changes in the functions of T-cells and memory. In contrast, knocking out ERK-2 is embryonic lethal (Saba-El-Leil *et al*, 2003). At present, the molecular basis for this diversification is unclear, as usually both MEK and ERK isoforms are coregulated. A potential answer could be provided by differential subcellular compartmentalisation and complex formation.

## SIGNAL DIVERSIFICATION THROUGH SCAFFOLDING PROTEINS AND SUBCELLULAR LOCALISATION

Signalling through this pathway is regulated by protein interactions that serve to connect activators with effectors, as well as target them to different subcellular localisations (Kolch, 2000). The binding of Raf kinases to Ras translocates Raf to the membrane compartment, where activation ensues. The artificial targeting to the plasma membrane suffices to partially activate Raf-1. Ha-Ras is not only activated at the cell membrane, but also on endomembranes, resulting in a differential interaction with downstream targets. Tethering Ha-Ras to the Golgi preferentially activated JNK, while Ha-Ras targeted to the endoplasmic reticulum stimulated ERK and Akt activities (Chiu *et al*, 2002). This observation suggests that the quality of the downstream signal is determined by the subcellular localisation of Ras, which by implication regulates the interaction with downstream effectors.

This theme is also encountered further downstream in the pathway. The scaffolding protein KSR constitutively binds to MEK. In response to mitogenic stimulation, the KSR/MEK complex is recruited from the cytosol to the cell membrane, where it can now interact with activated Raf-1 and ERK to facilitate the signal flux through the kinase module Raf → MEK → ERK (Muller *et al*, 2001). Gene knockout experiments in the worm *C. elegans*, which like mammals has two KSR genes, has revealed overlapping as well as some specific functions for the two KSR genes. However, when both genes were removed, ERK activation was severely compromised and the phenotype was similar to disabling the let-60 Ras gene (Ohmachi *et al*, 2002). Knocking out KSR1 in mice did not result in any gross abnormalities, although ERK activation in response to growth factors was modestly attenuated and the T-cell response to antigen was impeded. Remarkably, however, the KSR1^−/−^ mice were significantly less susceptible to developing mammary tumours when crossed to a transgenic tumour-prone strain (Nguyen *et al*, 2002). This opens a new opportunity for therapeutic intervention. Interfering with KSR expression or function would be expected to impede tumour growth, but leave normal cells unscathed.

**Figure 2:**
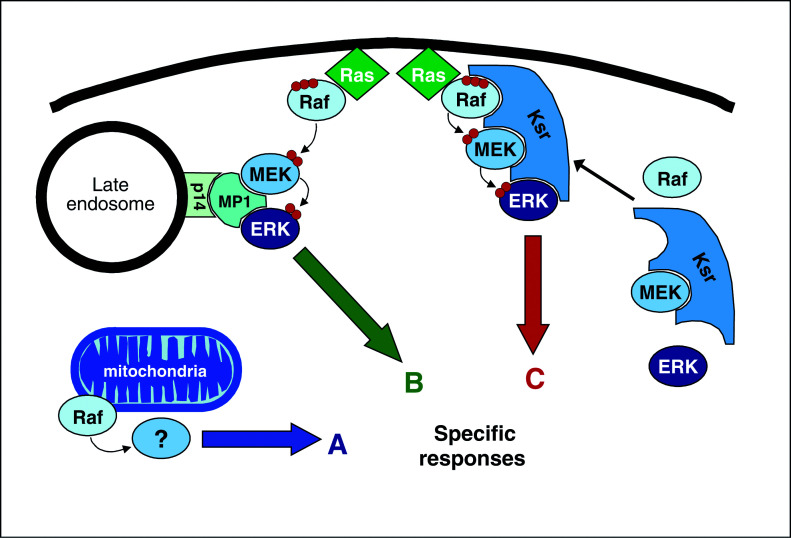
Different signalling complexes and subcellular compartmentalisation can generate diverse cellular responses. See text for details.

Another example is MP-1, a small scaffold that ties MEK and ERK together. MP-1 also binds to p14, an endosomal protein, which targets the MEK/ERK/MP-1 signalling complex to late endosomes. The downregulation of p14 or MP-1 protein levels diminished ERK activation and the induction of Elk-1-dependent reporter gene expression (Teis *et al*, 2002). These results suggest that the Raf/MEK/ERK pathway is organised in spatially distinct signalling complexes. It remains to be shown whether this correlates with functional diversity. An attractive possibility is that the spatial segregation could determine selective interactions with upstream activators and downstream effectors (Figure 2

).

For instance, a fraction of Raf-1 was found at the mitochondria and the artificial targeting of activated Raf-1 to mitochondria inhibited apoptosis. Curiously, mitochondrial Raf-1 did not activate the MEK-ERK pathway, but rather phosphorylated and inactivated BAD, a proapoptotic protein (Wang *et al*, 1996). Unfortunately, BAD was not confirmed as a direct Raf-1 substrate, and the search for the physiological substrate of mitochondrial Raf-1 is still ongoing. Interestingly, A-Raf was also detected in rat liver mitochondria. In contrast to Raf-1, which is loosely and peripherally associated, A-raf appears to be a true mitochondrial protein. It interacts with the putative mitochondrial import proteins hTOM and hTIM, and is found in the intermembrane space and the matrix (Yuryev *et al*, 2000). The function of A-Raf at the mitochondria is unknown. However, given that A-Raf is a very poor MEK kinase, an alternative mitochondrial substrate seems plausible.

These results also demonstrate that the identification of interaction partners is a viable strategy to unravel new functional connections. A recent systematic study used the N-terminal regulatory domains of Raf-1 and A-Raf, respectively, as baits in an exhaustive yeast two-hybrid screen (Yuryev and Wennogle, 2003). In total, 20 different proteins were identified, including several novel interaction partners. Half of these proteins exhibited isoform selectivity, six proteins binding to A-raf and four to Raf-1. Even more surprisingly, this selectivity was encoded by the cysteine-rich zinc-binding domain (CRD). This domain is essential for activation by Ras, although it is not the primary Ras-binding site (Avruch *et al*, 2001). These data pinpoint the CRD as a major hub for isoform selective protein interactions. They also predict the existence of a great number of distinct Raf signalling complexes, as the small size of the CRD makes simultaneous interactions unlikely. Clearly, a future challenge will be to unravel the composition of multiprotein signalling complexes *in situ* and characterise the dynamics of the interactions. The recent progress in proteomics and the improvement of imaging techniques for monitoring protein interactions at subcellular resolution and in real time will allow us to tackle these questions.

## CONCLUSION

The number of our genes is too small to account for the complexity of biological functions. Thus, the cell employs the same proteins in different contexts and imposes specificity through combinatorial mechanisms. Using the Raf/MEK/ERK signalling pathway as paradigm, we have highlighted some of these mechanisms including differential protein interactions, subcellular compartmentalisation, different modes of activation, and differential targeting of downstream effectors. A recent study elegantly demonstrates how the cell orchestrates this repertoire of mechanisms (Alavi *et al*, 2003). In endothelial cells, vascular endothelial growth factor (VEGF) protects from apoptosis caused by serum starvation and DNA-damaging drugs, whereas basic fibroblast growth factor (bFGF) prevents the apoptosis induced by death receptor stimulation. Both pathways employ Raf-1, yet use different modes of activation and different downstream effectors. bFGF protection is MEK independent and involves the activation of Raf-1 by phosphorylation of S338 and Raf-1 translocation to mitochondria. In contrast, VEGF-mediated survival is MEK dependent, does not translocate Raf-1 to mitochondria, and uses phosphorylation of Y341 to activate Raf-1. This example shows how the mode of activation could specify downstream signalling through differential subcellular localisation. Unveiling this combinatorial complexity will be the next great challenge for modern biology.

This task will also have enormous repercussions on drug development. Potent Raf and MEK inhibitors have been tested in clinical trials. In particular, the Raf inhibitor BAY 43-9006 was well tolerated, showing good efficacy as a single agent and in combination therapies (Hotte and Hirte, 2002). However, the realisation that one protein can have different functions in different cellular contexts will warrant new strategies for drug design. Target validation will rely on understanding these networks, which will only be possible at a systems biology level requiring quantitative biology combined with massive *in silico* simulation. We also will need to develop appropriate screens for new categories of targets, which may include protein interactions and subcellular distribution. Future drug development will rely on vigorous basic research in these areas and interdisciplinary collaboration to translate the findings into applications.
